# Bioactive Aromatic Plant Extracts Modulate Metabolism and Inflammation in HeLa Cells

**DOI:** 10.3390/molecules30224401

**Published:** 2025-11-14

**Authors:** Sara Silva, Manuela Machado, Manuela Pintado, Eduardo M. Costa

**Affiliations:** Universidade Católica Portuguesa, CBQF Centro de Biotecnologia e Química Fina-Laboratório Associado, Escola Superior de Biotecnologia, Rua Diogo Botelho 1327, 4169-005 Porto, Portugal; snsilva@ucp.pt (S.S.); mmachado@ucp.pt (M.M.); mpintado@ucp.pt (M.P.)

**Keywords:** aromatic plants, bioactive compounds, HeLa cells, phenolic compounds, anti-inflammatory activity, cytotoxicity, IL-6 modulation, metabolomics

## Abstract

Aromatic plants are rich sources of bioactive compounds with recognized therapeutic potential. This study investigated the phytochemical composition and biological activities of ethanolic extracts from four aromatic species—*Thymus vulgaris* L. (thyme), *Rosmarinus officinalis* L. (rosemary), *Aloysia citrodora* (lemon verbena), and *Tanacetum balsamita* L. (costmary)—using HeLa human cancer cells as a model. LC–MS analysis identified 28–44 metabolites per species, with phenolic compounds and terpenoids comprising 58–67% of total metabolites. Biological assays demonstrated concentration-dependent inhibition of HeLa cell metabolism down to 150 µg/mL, with rosemary displaying the strongest effects. LDH assays confirmed membrane disruption, most notably for lemon verbena (ca. 80% of release), and cellular proliferation was significantly disrupted by all extracts, most notably for thyme (70% reduction). Under oxidative conditions, costmary, thyme, and lemon verbena reduced intracellular ROS by up to 35% and all extracts suppressed IL-6 secretion, with rosemary showing the strongest anti-inflammatory response, lowering IL-6 levels to near or below the assay’s detection limit. Out of all the extracts, rosemary exhibited the most pronounced effects across cytotoxic, antioxidant, and cytokine assays, suggesting synergistic activity of its phenolic and terpenoid constituents. Multivariate analyses (correlation and PCA) linked specific metabolite classes to bioactivity patterns, providing insight into the mechanistic diversity underlying plant-specific effects. Overall, the results support the potential of these aromatic plants as sources of multifunctional bioactive compounds with anticancer and anti-inflammatory properties.

## 1. Introduction

Aromatic plants have long been used in traditional medicine for their therapeutic, aromatic, and nutritional properties. Ethnobotanical records report over fifty species historically employed to treat diverse ailments, reflecting their enduring value as sources of natural remedies [1]. Their popularity has grown in recent decades with increased interest in functional foods and bioactive natural products rich in phytochemicals [2,3].

The pharmacological potential of aromatic plants is largely attributed to their bioactive compounds such as polyphenols, terpenoids, and essential oils, which exhibit antioxidant, antimicrobial, and anti-inflammatory activities [4]. These bioactive constituents also contribute to neuroprotective and immunomodulatory effects, making aromatic plants promising candidates for the development of natural therapeutic agents [5,6]. Advances in analytical chemistry and metabolomics have enabled the identification and characterization of these compounds, supporting their use in pharmaceutical, nutraceutical, and food applications [7].

Recent research has highlighted the anticancer potential of aromatic plants. Extracts and essential oils displayed cytotoxic, anti-proliferative, and pro-apoptotic effects [8]. The activity of aromatic phytochemicals—particularly phenolics and terpenoids—is associated with the modulation of key signaling pathways, including NF-κB, MAPK, and JAK/STAT, and with the regulation of reactive oxygen species (ROS) and apoptotic responses [9]. Such mechanisms underline their value as multi-target compounds capable of influencing inflammation and tumor cell progression.

However, despite their therapeutic promise, potential risks remain, including toxic effects linked to phytochemical–enzyme interactions and heavy metal contamination, which may compromise safety and efficacy [10]. Therefore, controlled extraction and rigorous bioactivity testing are essential to ensure reproducibility and safety.

To evaluate their biological activity, HeLa cells were used as a model system. HeLa cells serve as a robust and reproducible platform for assessing the effects of bioactive compounds, owing to their well-characterized biology and responsiveness to diverse treatments [11]. This model has facilitated the discovery and mechanistic understanding of numerous potential anticancer agents, underscoring its importance in biomedical research [12]. Continued advancements in cellular and analytical technologies further enhance the utility of the HeLa model for exploring the mechanisms of action and therapeutic potential of natural bioactive compounds. Therefore, this study aimed to characterize the phytochemical composition of ethanolic extracts from *Thymus vulgaris*, *Rosmarinus officinalis*, *Aloysia citrodora*, and *Tanacetum balsamita* using LC–MS, and to evaluate their cytotoxic, antioxidant, and anti-inflammatory activities in HeLa cells. Through integrative metabolomic and functional analyses, correlations between metabolite classes and bioactivity were explored to elucidate their underlying mechanisms.

## 2. Results and Discussion

### 2.1. Phenolic Compounds Fingerprinting

**Figure 1:**
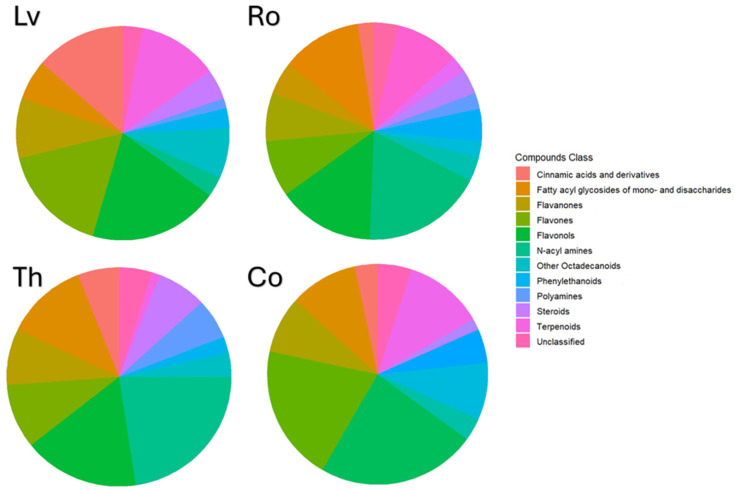
Relative distribution of chemical classes identified in the extracts of four plant species. Pie charts illustrate the proportion of metabolite classes detected by LC–MS analysis in lemon verbena (Lv), rosemary (Ro), thyme (Th), and costmary (Co). Each color represents a distinct compound class as indicated in the legend.

The full fingerprint characterization of the extracts through LC-MS is presented in the Supplementary Materials (Table S1). Twenty-eight individual compounds were identified for lemon verbena (Lv) and thyme (Th), 44 for rosemary (Ro) and 38 for costmary (Co), with the main chemical classes represented in Figure 1 and distribution per class described in Table 1. From Table 1, it is evident that phenolic compounds classes comprised the majority of extracted compounds, representing 58.4% (Lv), 59.0%(Ro), 60.7%(Th) and 66.6% (Co) of total metabolites. Through Area Under the Curve (AUC) calculations using chlorogenic acid as a standard, these values corresponded to average Total Phenolic Content (TPC) values of 7.73 ± 0.0425 g Chlorogenic Acid Equivalents (CAE)/100 g of extract for thyme, 7.04 ± 0.101 g Chlorogenic Acid Equivalents (CAE)/100 g of extract for rosemary, 30.67 ± 1.407 g Chlorogenic Acid Equivalents (CAE)/100 g of extract for costmary and 2.56 ± 0.331 g Chlorogenic Acid Equivalents (CAE)/100 g of extract for lemon verbena. These results and the identified compounds align with previous works of Pereira et al. [13] for thyme, Pereira, et al. [14] for lemon verbena, Nie et al. [15] for rosemary and Gevrenova et al. [16] for costmary.

### 2.2. Impact upon Cellular Metabolism

**Figure 2:**
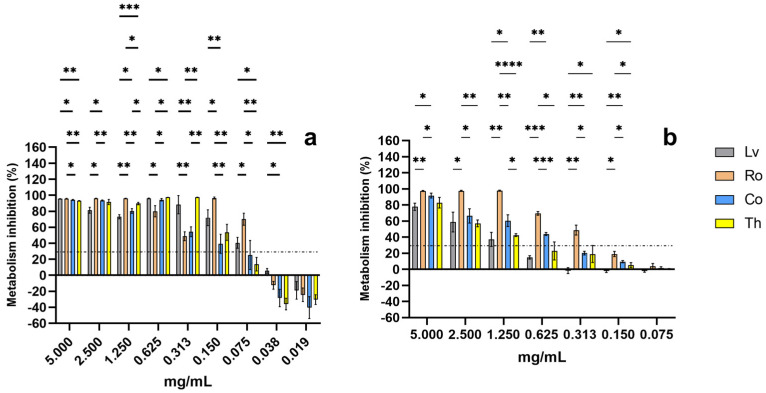
Impact of aromatic plants extracts upon Hela (**a**) and Vero (**b**) cellular metabolism. Dotted line represents the 30% cytotoxicity limit as defined in the ISO 10993-5: 2009 [17]. Data are presented as mean ± SD (*n* = 4). * Represents a 95% statistical significance (*p* < 0.05), ** represents 99% statistical significance (*p* < 0.01), *** represents 99.9% statistical significance (*p* < 0.001) and **** represents 99.99% statistical significance (*p* < 0.0001).

The impact of the evaluated extracts on the selected cell lines is shown in Figure 2.

For Vero cells (Figure 2b), the highest concentration at which extracts presented no cytotoxic effects was 0.150 mg/mL. Coincidentally, this was also the lowest concentration at which all extracts exhibited cytotoxic effects on HeLa cells (Figure 2a). Across all tested concentrations—excluding the lowest levels for both lines—the extracts displayed statistically significant differences (*p* < 0.05). Among them, rosemary (Ro) demonstrated the highest metabolic inhibition in both cell lines.

The results for thyme (Th) align with previous studies. Oliveira et al. [18] observed that an aqueous commercial thyme extract did not significantly reduce HeLa cell viability up to 50 mg/mL—considerably higher than the values observed here—likely due to differences in solvent polarity and extract composition. In contrast, Berrington and Lall [19] reported that an acetone thyme extract exhibited IC_50_ values above 0.2 mg/mL for HeLa and 0.138 mg/mL for Vero cells, which differ from those observed in this study. Heidari et al. [20], while not against HeLa, also demonstrated the cytotoxicity of an ethanolic thyme extract towards T47D human breast cancer cells at 0.2 mg/mL, consistent with our findings. Similarly, N. Adham et al. [21] reported strong cytotoxic effects of thyme extracts against leukemia cell lines at concentrations as low as 0.0021–0.094 mg/mL, while non-cancerous peripheral blood mononuclear cells remained unaffected up to 0.1 mg/mL, suggesting a selective cytotoxic behavior comparable to the one observed here.

For rosemary (Ro), the findings here observed are consistent with the work of Nie, Li, Wang, Tan, Tang and Jiang [15], who reported significant inhibition of HeLa cell metabolism by ethanolic rosemary extracts from 0.5 mg/mL onwards. However, Gonçalves et al. [22] observed that a crude aqueous rosemary extract was not cytotoxic toward HeLa cells up to 0.4 mg/mL, highlighting the strong influence of solvent polarity on extract bioactivity. The lower values observed here likely reflect the higher concentration of bioactive compounds (e.g., terpenoids and phenolics) in the ethanolic extract.

Regarding costmary (Co) no previous work exists on the topic. However, Sharma et al. [23] reported other *Tanacetum* species (*Tanacetum dolicophyllum*) as having an IC_50_ of 0.075 mg/mL against HeLa cells, a value equal to the one here observed. Furthermore, Gevrenova, Zengin, Sinan, Zheleva-Dimitrova, Balabanova, Kolmayer, Voynikov and Joubert [16] previously reported that a methanolic costmary extract reduced THP-1 (human monocytic) cell viability by 50% at 0.2 mg/mL, which is consistent with our findings and supports the presence of cytotoxic secondary metabolites in this species, such as sesquiterpene lactones and phenolic compounds.

For lemon verbena (Lv), previous works have shown that ethanolic extracts of this plant were not cytotoxic towards Vero cells up to 0.625 mg/mL while simultaneously showing a strong inhibitory effect upon tumoral cell lines, a result equal to the one observed here [24].

As the lowest concentration at which all extracts presented significant metabolism inhibition (without deleterious effects in Vero cells) was 0.150 mg/mL it was selected to be used in the following assays.

### 2.3. Membrane Integrity

Targeting the cell membrane integrity is a promising strategy for anti-tumoral approaches due to its potential to disrupt cellular functions and induce cell death and is among the emerging strategies for therapeutic approaches [25,26].

**Figure 3:**
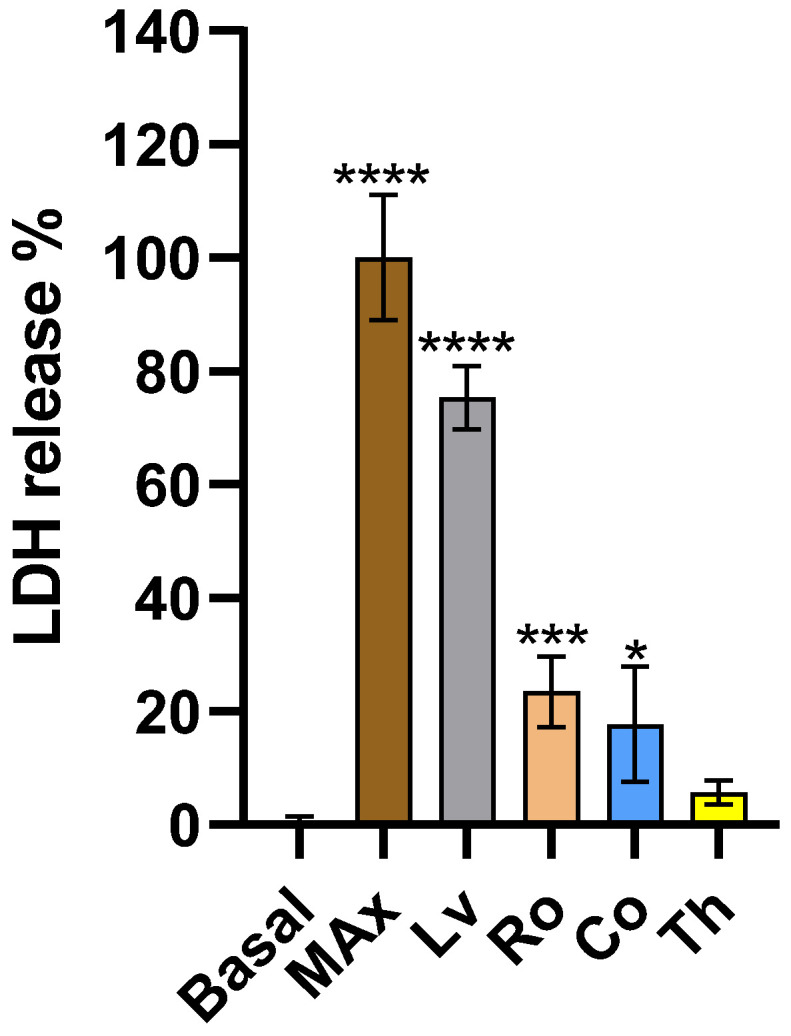
Impact of aromatic plants extracts upon HeLa membrane integrity. Max corresponds to Maximum LDH release using the kits lysis buffer. Data are presented as mean ± SD (*n* = 4). Statistical differences were analyzed using one-way ANOVA followed by Tukey’s post hoc test. *p* < 0.05 (*), *p* < 0.01 (***), *p* < 0.0001 (****).

As can be seen from Figure 3, the evaluated extracts at the selected concentration all led to increased lactate dehydrogenase (LDH) release, indicating interactions with and damage of the cellular membrane. Of the evaluated extracts, Lv presented statistically significant (*p* < 0.001) higher release percentage than all other evaluated conditions. Except for Th, all extracts presented LDH release percentages significantly higher (*p* < 0.05; *p* < 0.001 and *p* < 0.0001) than that of the basal control.

Comparison of these results with the literature is difficult as little to no work is available on this topic regarding either the used plants or targeted cell line. The only work remotely on the topic is the one on HeLa by Ayesh et al. [27] in which a ethanolic extract of Th had no effect on membrane integrity of THP-1 cells up to 20 mg/mL. However, this must be considered carefully as THP-1 cells are non-adherent and thus present different compound susceptibility.

### 2.4. Proliferation

**Figure 4:**
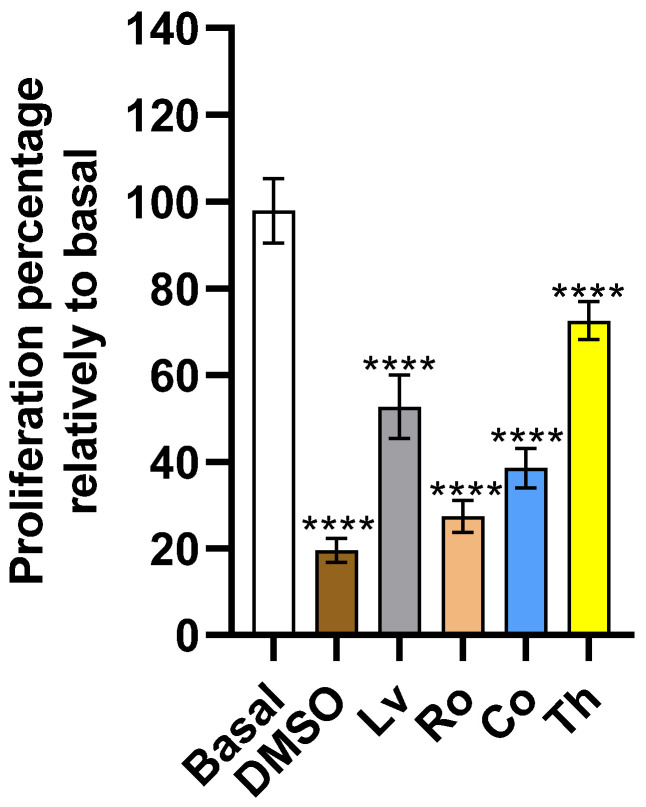
Impact of aromatic plants extracts upon Hela cell proliferation. Results are expressed as a percentage relative to the basal condition (untreated control). Negative control corresponds to the treatment with DMSO. Data are presented as mean ± SD (*n* = 4). Statistical significances were analyzed using one-way ANOVA followed by Tukey’s post hoc test. *p* < 0.0001 (****).

Cellular proliferation is critical in tumoral development and progression and its targeting a valuable and tested solution to halt cellular expansion [28]. The data obtained (Figure 4) showed that all extracts were effective in reducing HeLa proliferation with statistically significant (*p* < 0.0001) reductions relative to the basal condition being observed. Between extracts, statistically significant (*p* < 0.01) differences were observed with the highest reduction being observed for Ro and the lowest for Th.

The results obtained for lemon verbena (Lv) are consistent with previous findings reported in the literature. Pereira, Pimenta, Calhelha, Antonio, Barros, Santos-Buelga, Verde and Ferreira [14] demonstrated that a phenolic-rich Lv extract exhibited marked anti-proliferative activity against HeLa cells at concentrations between 0.225 and 0.249 mg/mL. Similar results were later described by Pereira et al. [29], who reported proliferation inhibition values ranging from 0.232 to 0.249 mg/mL, confirming the inhibition potential of Lv extracts within a comparable concentration range. Although not for HeLa cells, Rashid, Mahmod, Afifi and Talib [24] showed that Lv inhibited tumoral cell proliferation at concentrations between 0.625 and 1.25 mg/mL, values higher than those observed here.

For thyme (Th), the findings are likewise in line with earlier studies. Pereira, Pimenta, Barros, Calhelha, Antonio, Cabo Verde and Ferreira [29] reported inhibition of HeLa cell proliferation at concentrations between 0.204 and 0.228 mg/mL, while Pereira, Pimenta, Calhelha, Antonio, Verde, Barros, Santos-Buelga and Ferreira [13] described slightly lower inhibitory concentrations, between 0.160 and 0.191 mg/mL, suggesting a strong dependence on extract composition and phenolic content. These values correspond well with those observed in the present study, supporting the reproducibility of thyme’s anti-proliferative activity across different extraction protocols.

For rosemary (Ro), the observed inhibitory effects on HeLa cell proliferation are supported by the work of Đilas et al. [30], who showed that commercial rosemary extracts significantly reduced HeLa cell viability from concentrations as low as 0.002 mg/mL. This pronounced activity underscores the potency of rosemary-derived compounds, likely related to the presence of diterpenes and phenolic acids with known cytotoxic properties.

For costmary (Co), while no data dealing directly with the assayed plant exist, existing data from other *Tanacetum* species extracts report inconsistent anti-proliferative activity on HeLa cells, contrary to the results observed here [23].

### 2.5. Cellular Antioxidant Activity

Cellular redox homeostasis is crucial for genomic integrity and cell signaling, with imbalances in redox status leading to uncontrolled cell proliferation [31]. Thus, monitoring compound capacity to alter this balance is of the utmost importance.

**Figure 5:**
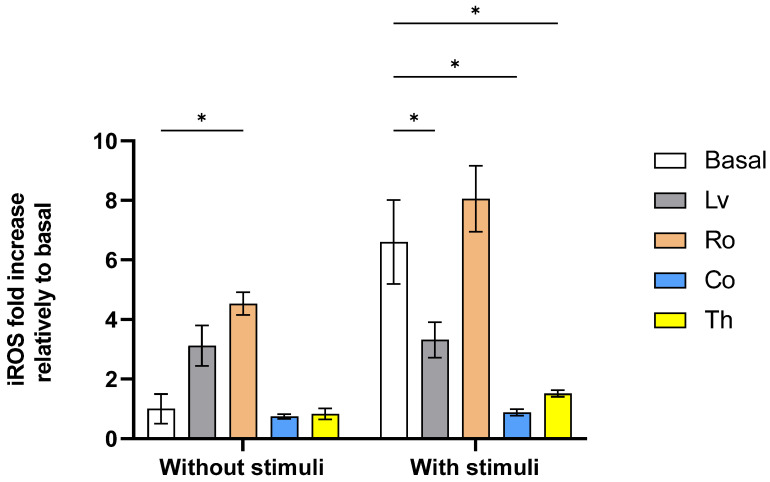
Impact of aromatic plants extracts upon HeLa cellular antioxidant activity. Data are presented as mean ± SD (*n* = 4). Statistical analysis was performed using one-way ANOVA followed by Tukey’s post hoc test. *p* < 0.05 (*).

The data obtained (Figure 5) showed that regardless of the presence or absence of a pro-oxidant stimulus, Ro always led to increases in iROS levels, with this increase being statistically significant (*p* < 0.05) when no stimulus was present. Lemon verbena (Lv) also presented an increase in iROS production without stimuli, but this increase was not statistically significant (*p* > 0.05). When a pro-oxidant stimulus was added to the system Lv, Co and Th presented statistically significant (*p* < 0.05) reductions in iROS levels relative to the stimulated basal control, with the strongest effect being observed for costmary (Co). These results are largely in line with the existing literature, as extracts rich in phenolics and flavonoids have been shown to be capable of scavenging ROS, upregulating antioxidant enzymes (SOD, CAT, GPx), and modulating redox-sensitive transcription factors (e.g., Nrf2, NF-κB) [32,33].

For rosemary (Ro), the data obtained here represents an outlier, as Nie, Li, Wang, Tan, Tang and Jiang [15] previously reported that the Ro ethanolic extract reduced iROS in HeLa cells stimulated with H_2_O_2_, with this activity linked with the enhancement of the antioxidant enzymes superoxide dismutase and catalase [34,35]. A possible explanation is that this may be a context-dependent atypical response as previously reported by Valdés et al. [36].

For thyme (Th), no previous data correlating this plant ethanolic extracts and HeLa cells is available. However, related studies have shown that in cancerous cells (plasmacytoma myeloma and neuroblastoma cells), Th had pro-oxidant activity as it increased iROS levels [21,37]. On the other hand, Th extracts have been described as being capable of reducing iROS levels in murine models and in HepG2 cells, with this activity being related to the promotion of superoxide dismutase and glutathione peroxidase enzyme activity [38,39].

Regarding lemon verbena (Lv), as with Th, there are no previous works on this topic directly relating ethanolic extracts of this plant to HeLa cells. Nevertheless, the data reported here is in line with the activity reported in other cell lines [16]. Coincidently, as with thyme, Lv and Ro activity has been linked to the enhancement of antioxidant enzymes such as catalase, glutathione peroxidase, and glutathione reductase [40,41].

Lastly, for costmary (Co), there is no previous data on the topic and even on possible application of this plant in iROS modulation. Thus, the observed data may only be explained by the rich profile of secondary metabolites, including phenolic compounds and flavonoids, which are known for their antioxidant properties [16].

### 2.6. IL-6 Secretion Modulation

**Figure 6:**
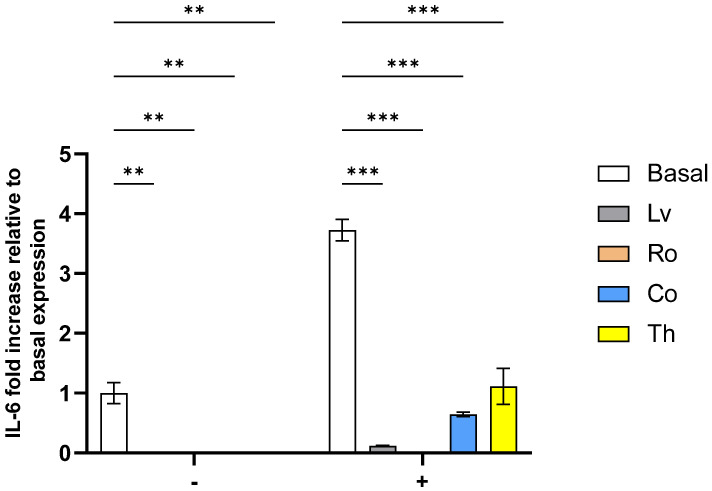
Effect of aromatic plants extracts upon Hela IL-6 secretion in the presence (+) or absence (-) of an inflammatory stimulus. Results are expressed as fold increase relative to basal (unstimulated and untreated) expression. Data are shown as mean ± SD (*n* = 4). Statistical significances were determined using one-way ANOVA followed by Tukey’s post hoc test. *p* < 0.01 (**), *p* < 0.001 (***).

In the HeLa tumoral process, interleukin-6 (IL-6) plays a crucial role in promoting tumor growth, proliferation, and even resistance to therapy. Therefore, assessing the effects of plant extracts on IL-6 production is of particular biological importance [42]. The data obtained (Figure 6) demonstrate that all extracts exerted a strong inhibitory effect on IL-6 secretion, with statistically significant reductions (*p* < 0.01; *p* < 0.001) observed across treatments. Under basal, non-stimulated conditions, IL-6 levels in the presence of the extracts were below the assay’s quantification limit (4 pg/mL), confirming an absence of pro-inflammatory activity. Upon inflammatory stimulation, IL-6 secretion in the basal control increased markedly, validating activation of the inflammatory pathway. More importantly, treatment with the extracts significantly counteracted this effect, confirming the potential anti-inflammatory activity, with Ro standing out as the best performer, followed by Lv.

Regarding the anti-inflammatory potential observed here, there are no previous works on this topic for the plants assayed. However, these plants have already demonstrated anti-inflammatory potential in different settings. Thyme (Th) has been shown to reduce cytokines secretion (including IL-6) in stimulated murine macrophages, splenocytes, and bronchial and tracheal epithelial cells [18,43,44]. A possible explanation for this activity may be found in the work of Oliviero, Romilde, Beatrice, Matteo, Giovanna, Consuelo, Claudio, Giorgio, Maggi and Massimo [44], which showed that Th extracts down-regulate NF-κB signaling, a pathway critical for pro-inflammatory cytokine production.

For lemon verbena (Lv), anti-inflammatory activity has been previously reported towards A549 cells (lung carcinoma) and even in the serum of multiple sclerosis patients [45,46], with this activity being ascribed to the presence of the compound verbascoside (also known as acteoside), which is present in the sample assayed (Table S1, Supplementary Materials) and has been shown to interfere with various inflammation-associated pathways, including JAK/STAT signaling.

For rosemary (Ro), as with Lv, indirect inferences can be made as extracts have been shown to reduce IL-6 secretion in Caco-2 (intestinal) cells, mouse macrophages and in murine models [34,47,48]. This activity has been mainly ascribed to the presence of rosmarinic acid, which is present in the sample assayed (Table S1, Supplementary Materials), and thought to be related to inhibition of the NF-KB and MAPK pathways, which are both crucial for IL-6 transcription [44,49].

Lastly, for costmary (Co), even data detailing any possible anti-inflammatory effects does not exist. However, other *Tanacetum* species have shown capacity to inhibit IL-6 secretion in human macrophages and microglia cells [50,51], thus lending some credence to the data observed here.

### 2.7. Integrative Data Analysis

**Figure 7:**
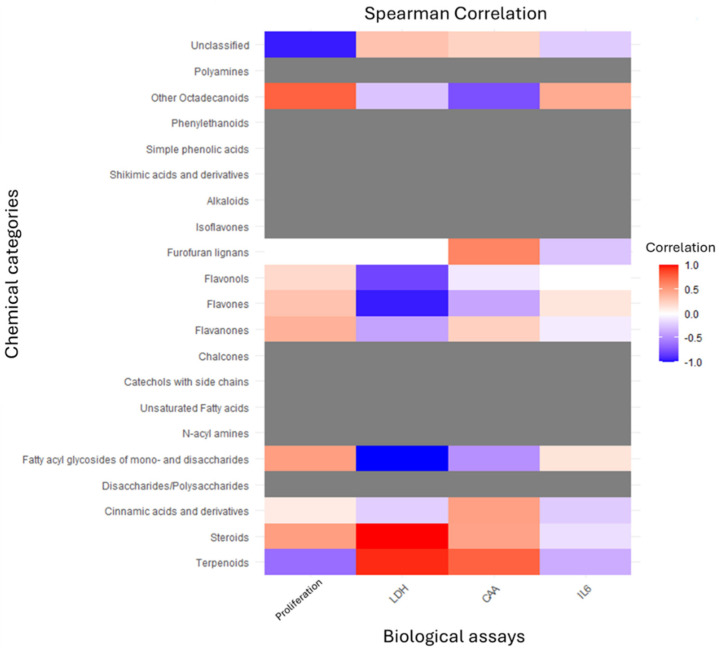
Spearman correlation heat map between chemical classes and biological responses. The heat map depicts the correlation coefficients (ranging from −1 to +1) between the relative abundance of chemical classes identified by LC–MS and the measured biological parameters: cell proliferation, lactate dehydrogenase (LDH) release, cellular antioxidant activity (CAA), and interleukin-6 (IL6) levels. Positive correlations (red) indicate a direct relationship, whereas negative correlations (blue) denote inverse associations.

The heat map (Figure 7) provides a comprehensive visualization of the relationships between chemical classes and biological responses, illustrating the complexity of the metabolite–bioactivity interplay. The correlation patterns reveal that the biological effects of the analyzed plant extracts are strongly influenced by their predominant classes of secondary metabolites. Among these, steroids exhibited a strong positive correlation with LDH, indicating their potential contribution to cytotoxic effects, likely through membrane disruption or metabolic interference. In contrast, fatty acyl glycosides of mono- and disaccharides showed a strong negative correlation with LDH, suggesting a possible protective role against cellular damage, potentially due to membrane-stabilizing or antioxidant properties.

Flavonoids, including flavones, flavonols, and flavanones, demonstrated moderate positive correlations with cell proliferation, consistent with their reported roles in promoting cell viability, modulating signaling pathways, and supporting tissue regeneration. Conversely, unclassified compounds correlated negatively with proliferation, suggesting the presence of bioactive molecules with inhibitory or anti-proliferative potential. Furthermore, terpenoids and other octadecanoids showed moderate positive associations with cellular antioxidant activity, reinforcing their contribution to redox balance and defense against oxidative stress. Overall, these findings emphasize that different chemical classes exert distinct biological effects, reflecting the structural diversity of metabolites and their potential synergistic or antagonistic interactions. Such correlation-based insights provide a valuable framework for linking phytochemical composition to bioactivity, guiding future isolation and mechanistic studies aimed at identifying key bioactive constituents responsible for the observed effects.

**Figure 8:**
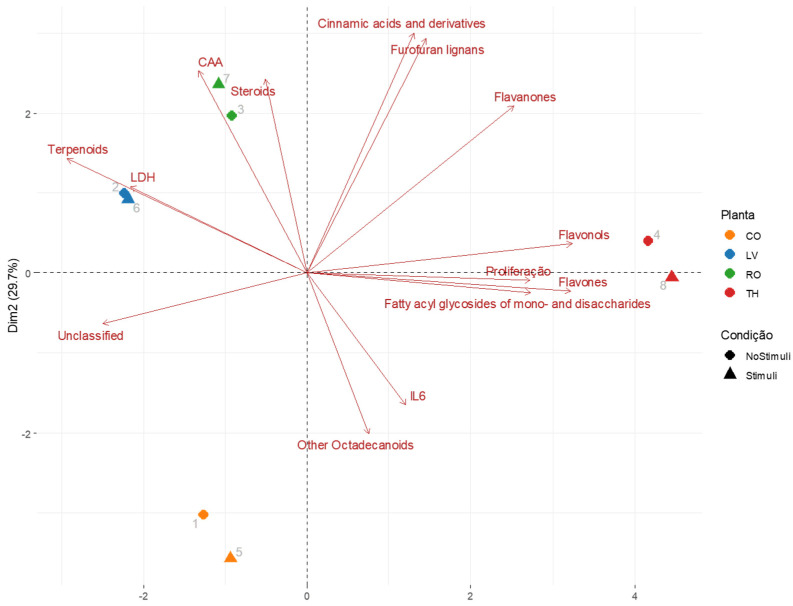
Principal component analysis (PCA) biplot integrating chemical classes and biological responses across four plant species. The PCA illustrates the distribution of costmary (orange), lemon verbena (blue), rosemary (green), and thyme (red) according to their metabolite composition and associated biological responses. Red vectors represent chemical classes, while black arrows indicate biological variables (cell proliferation, LDH, CAA, and IL6).

The principal component analysis (PCA) (Figure 8) provided a detailed multivariate assessment of the relationships between the chemical composition of the plant extracts and their biological activities, allowing for the identification of discriminant metabolite classes among the four species analyzed. The first two principal components (PC1 and PC2) together accounted for 78.3% of the total variance (57.4% and 20.9%, respectively), reflecting a robust model with high explanatory power. This dimensional reduction enabled a clear separation of costmary, lemon verbena, rosemary, and thyme based on their metabolite profiles and associated bioactivities.

The distribution of samples revealed that rosemary (Ro) clustered towards the upper-left quadrant, closely associated with steroids, which strongly aligned with the cellular antioxidant activity (CAA) vector. This suggests that steroidal constituents, potentially including phytosterols and related triterpenoids, are key contributors to the antioxidant potential of rosemary, possibly through the stabilization of cellular membranes and modulation of redox homeostasis.

Thyme (Th) samples, positioned on the right-hand side of the plot, were associated with flavonoid classes—notably flavones, flavonols, and flavanones—which correlated positively with cell proliferation. This association is consistent with the established role of flavonoids in promoting cellular growth and protecting against oxidative stress, as well as their involvement in signaling pathways that regulate cell survival.

In contrast, lemon verbena (Lv) samples, located in the lower-left quadrant, were strongly associated with terpenoids and the LDH vector. This indicates that the terpenoid-rich profile of this species may contribute to higher LDH release, suggesting potential cytotoxic or membrane-disruptive effects. Such activity could stem from the lipophilic nature of terpenoids, which allows them to interact with and perturb cellular membranes or induce oxidative stress.

Costmary extract (Co) was positioned near other octadecanoids and unclassified compounds, showing an inverse relationship with proliferation and IL6 levels. This pattern may indicate the presence of anti-proliferative or anti-inflammatory metabolites, such as fatty acid derivatives, which could modulate cytokine responses and cellular metabolism.

Overall, the PCA biplot demonstrates that distinct chemical classes underpin the biological profiles of each plant species. Rosemary and thyme, characterized by steroidal and flavonoid constituents, respectively, exhibited associations with antioxidant and proliferative responses, while lemon verbena and costmary, enriched in terpenoids and octadecanoid-like compounds, were linked to cytotoxic and regulatory effects. These findings align with the correlation analysis, reinforcing that metabolite composition exerts a decisive influence on biological outcomes. This integrative approach highlights the value of multivariate analysis in linking chemical diversity to functional bioactivity, providing a mechanistic basis for the observed species-specific effects.

## 3. Materials and Methods

### 3.1. Chemicals and Raw Materials

Ethanol, formic acid and chlorogenic acid standard used in this work were of analytical grade and attained from Sigma (Sigma-Aldrich, St. Louis, MO, USA). HPLC and LC-MS solvents were attained pre-prepared from VWR (West Chester, PA, USA). Lemon verbena (Lv, *Aloysia citrodora*), thyme (Th, *Thymus vulgaris*), rosemary (Ro, *Salvia rosmarinus*) and costmary (Co, *Tanacetum balsamita*) were harvested from an open-field cultivation at the end of the production cycle in September 2023 from a local farm in Sanguedo, Vila Nova de Gaia, Portugal. The collected plant material was vacuum-sealed and stored at −20 °C until further analysis.

### 3.2. Compound Extraction

Extracts were prepared at 10% (m/v) using EtOH and left to react overnight at room temperature. Samples were then centrifuged with supernatants being recovered and solvents evaporated via rotary evaporation (Büchi, Flawil, Switzerland). The resulting solid was then collected and stored under hygroscopic conditions until use. For all biological assays, samples were reconstituted in EtOH.

### 3.3. High-Resolution LC-MS and Data Analysis

High-resolution LC-MS analysis was performed following the method described by Machado et al. [52]. The analysis employed an LC–ESI–UHR–QqTOF–MS system (Impact II, Bruker, Billerica, MA, USA) coupled to an UltiMate 3000 Dionex ultra-high-performance liquid chromatography unit (UHPLC, Thermo Scientific, Waltham, MA, USA). Data acquisition was carried out in negative ionization mode. Metabolite separation was achieved using an Acclaim RSLC 120 C18 column (100 × 2.1 mm, 2.2 μm; Dionex, Sunnyvale, CA, USA). The mobile phases consisted of 0.1% formic acid in water (solvent A) and acetonitrile containing 0.1% formic acid (solvent B). The gradient elution program began at 5% B, increased linearly to 95% B over 7 min, was maintained at 95% B for 2 min, returned to 5% B within 1 min, and was held at 5% B for a further 5 min. The flow rate was set at 0.25 mL/min, with an injection volume of 5 μL.

Mass spectrometric data were collected in positive ionization mode over a mass range of m/z 20–1000. The MS parameters were as follows: capillary voltage, 4.5 kV; drying gas temperature, 200 °C; drying gas flow rate, 8.0 L/min; nebulizing gas pressure, 2 bar; collision RF, 300 Vpp; transfer time, 120 μs; and pre-pulse storage time, 4 μs. Internal mass calibration was performed post-acquisition using sodium format clusters, delivered via a syringe pump at the beginning of each chromatographic run.

Data analysis was conducted using MZmine 4.4.3 for mass detection, chromatogram normalization, deconvolution, and alignment. The detected compounds were identified as metabolites based on retention time (t_0_), accurate molecular mass, predicted molecular formula, and MS/MS fragmentation patterns, by comparison with publicly available databases (e.g., GNPS and PubChem). The MZmine-generated feature table, containing aligned retention times, m/z values, and peak areas, was further processed through the Global Natural Products Social Molecular Networking (GNPS) online workflow. Compound annotation was performed using the GNPS library matching tool, applying the following parameters: precursor mass tolerance of 2.0 Da, fragment ion tolerance of 0.5 Da, cosine score threshold of 0.7, and a minimum of six matching fragment peaks.

### 3.4. Cell Lines Used

Hela (ATCC CCL-2) and Vero cells (ATCC CCL-81) were cultured as monolayers using Dulbecco’s modified Eagle’s medium (DMEM) with 4.5 g/L glucose, L-glutamine without pyruvate (ThermoScientific, MA, USA) containing 10% fetal bovine serum (ThermoScientific, MA, USA) and 1% (*v*/*v*) Penicillin–Streptomycin–Fungizone (ThermoScientific, MA, USA). Cells were cultured at 37 °C in a humidified atmosphere of 95% air and 5% CO_2_ and used between passages 33 and 40.

### 3.5. Impact upon Cellular Metabolism

HeLa cell viability was assessed following the ISO 10993-5:2009 standard, as previously described by Costa et al. [53]. Briefly, cells were cultured to 80–90% confluence, detached, and seeded at a density of 1 × 10^5^ cells/mL in 96-well microplates. After 24 h, the medium was replaced with fresh medium containing plant extracts at concentrations ranging from 0.0188 to 5 mg/mL. Plain medium and medium containing 30% (*v*/*v*) DMSO were used as growth and death controls, respectively. After 24 h, 10 µL of PrestoBlue (Thermo Fisher Scientific, MA, USA) were added to each well and the plates re-incubated. After 1 h, fluorescence (Ex: 560 nm; Em: 590 nm) was measured using a microplate reader (Synergy H1, Biotek Instruments, Winooski, VT, USA). All assays were performed in quadruplicate and results were given in terms of percentage of cell metabolism inhibition.

### 3.6. Impact upon Cell Membrane Integrity

The samples’ impact upon Hela cell membrane integrity was evaluated as previously described by Costa et al. [54]. Briefly, cells were seeded as described in Section 2.5, and after 24 h, exposed to compounds at the highest non-cytotoxic concentration. As a control plain media was used. After 24 h, the extracellular levels of LDH were evaluated using the CyQuant LDH cytotoxicity assay (ThermoFisher Scientific, MA, USA) according to the manufacturer’s instructions. All samples were evaluated in quadruplicate and results were given in terms of percentage of LDH release.

### 3.7. Impact upon Cellular Proliferation

Evaluation of samples effect upon Hela proliferation was evaluated using the Cyquant Direct Cell Proliferation assay kit (Thermo Fisher Scientific, Waltham, MA, USA) as previously described by Machado, Silva, Pintado and Costa [52]. Briefly, cells were seeded at 1 × 10^5^ cells/mL in a 96-well microplate, and after 24 h, were exposed to phenolic extracts at concentrations without deleterious metabolic effects. After 24 h of exposure, the kit’s cell-permeant DNA-binding dye was added to the wells, the plate re-incubated for 1 h, and then fluorescence (Ex: 480 nm; Em: 535 nm) was measured using a microplate reader. All assays were performed in quadruplicate and results calculated according to the manufacturer’s instructions.

### 3.8. Impact upon Intracellular ROS Production

Evaluation of samples effect upon Hela’s iROS production was evaluated using the Cellular ROS Assay Kit from Abcam (ab113851, Cambridge, UK) through adaptation of the work of Costa et al. [55]. Briefly, cells were seeded 2.5 × 10^5^ cells/well in a black, 96-well microplate (Nunc Nunclon, ThermoScientific, Roskilde, Denmark) and incubated at 37 °C with 5% CO_2_. After 24 h, wells were washed with PBS and samples with or without iROS inducer (TBHP) were added to the wells and the plate was re-incubated for 4 h. Plain media and TBHP at 0.50 µM were used as basal control and positive control, respectively. iROS formation was measured by fluorescence (Ex:495 nm; Em: 529 nm) using a microplate reader. All determinations were performed in quadruplicate and data was given in terms of fold expression increase or decrease relative to basal levels expression.

### 3.9. Modulation of IL-6 Production

Evaluation of samples capacity to inhibit IL-6 expression in Hela cells was performed through adaptation of the work of Yano et al. [56]. Briefly, Hela cells were seeded at 1 × 10^5^ cells/well in 24-well plates and incubated overnight. Samples at non-cytotoxic concentrations were added in conjunction with TNF-α at 5 ng/mL (Peptrotech, Cranbury, NJ, USA) and re-incubated. Cells with plain media were used as basal control while cells with media and TNF- α were used as inflammation control. After 24 h, supernatants were recovered and frozen at −80 °C for posterior analysis. All assays were performed in quadruplicate.

Target interleukin detection and quantification was performed via ELISA assays using the ELISA MAX Deluxe Set Human IL-6 (BioLegend, San Diego, CA, USA) according to the manufacturer’s instructions. All determinations were performed in quadruplicate and data given in terms of fold expression increase or decrease relative to basal levels expression.

### 3.10. Statistical Analysis

The statistical analysis of the data was performed using Prism 9 for Windows (GraphPad Software, Boston, MA, USA). As the data followed a normal distribution, results were analyzed using one-way ANOVA followed by Tukey’s post hoc test. Differences were considered statistically significant at *p* < 0.05.

Multivariate analyses were carried out using R software (version 4.5.0; R Core Team, 2025). Data was normalized by z-score transformation prior to multivariate and correlation analyses. The relationship between chemical categories and biological activities (cell proliferation, LDH release, CAA, and IL-6) was assessed using Spearman’s rank correlation via the cor() function from the stats package. Correlation coefficients (ρ) with |ρ| ≥ 0.5 were considered biologically relevant. Results were visualized as a heat-map using the ggplot2 and reshape2 packages, with color gradients representing the strength and direction of correlations. A principal component analysis (PCA) was subsequently performed to identify clustering patterns among samples and to visualize the association between chemical profiles and bioassay responses. The analysis was conducted using the prcomp() function from the stats package with centered and scaled data. The PCA biplot was generated with ggplot2, where loading vectors represented chemical categories contributing most to sample separation. The proportion of variance explained by each principal component was used to interpret the underlying data structure. All graphical outputs were customized using ggplot2 and auxiliary packages (grid, ggrepel, and scales) for improved clarity and visualization quality.

## 4. Conclusions

This study provides a comprehensive assessment of the phytochemical and biological profiles of four aromatic plant extracts—thyme, rosemary, lemon verbena, and costmary—highlighting their cytotoxic, antioxidant, and anti-inflammatory activities in HeLa cells. LC–MS-based metabolomic profiling revealed that phenolics and terpenoids were the main bioactive classes present and thus probably responsible for these effects. All extracts significantly inhibited cell proliferation and IL-6 secretion, underscoring their potential to modulate tumor-related inflammation. Rosemary emerged as the most active extract, displaying strong inhibitory effects on both oxidative stress and cytokine production, while costmary demonstrated previously unreported bioactivity. Integrating metabolomic and functional analyses enabled the identification of chemical–biological correlations, suggesting distinct mechanistic pathways for each species. Collectively, these findings reinforce the relevance of aromatic plants as promising sources of natural compounds with anticancer and anti-inflammatory potential, warranting further investigation toward compound isolation and mechanistic validation.

## Figures and Tables

**Table 1:** Main compounds class present in the extracts of each aromatic plant. Values represent the percentage of compounds identified per class. “Unclassified” corresponds to compounds that were not identified.

Compound Class\Plant	Lv	Ro	Th	Co
Alkaloids	-	2.4	-	-
Catechols with side chains	-	-	6	-
Chalcones	-	-	-	3.3
Cinnamic acids and derivatives	13.6	12.0	11.9	10
Disaccharides/olysaccharides	-	4.8	-	-
Fatty acyl glycosides of mono- and disaccharides	6.1	7.2	8.3	-
Flavanones	9.1	8.4	9.5	8.3
Flavones	16.7	14.5	16.7	20
Flavonols	19.7	18.1	22.6	23.3
Furofuran ligans	-	3.6	3.6	3.3
Isoflavones	-	2.4	-	-
N-acyl amines	3.0	-	2.4	-
Other octadecanoids	7.6	4.8	6	8.3
Phenylethanoids	3.0	-	-	-
Polyamines	1.5	-	-	-
Steroids	4.5	-	-	-
Shikimic acids and derivatives	-	2.4	-	-
Simple phenolic acids	-	3.6	-	5
Steroids	-	2.4	-	1.7
Terpenoids	12.1	9.6	7.1	11.7
Unclassified	3.0	3.6	1.2	5
Unsaturated fatty acids	-	-	4.8	-

## Data Availability

The data presented in this study are available on request from the corresponding author. The data are not publicly available due to confidentiality agreements.

## Supplementary Materials

The following are available online at https://www.mdpi.com/article/10.3390/molecules30224401/s1, Table S1: Compounds identified by LC-MS for the ethanolic extracts of the different aromatic plants evaluated.
