# Supplementary material for: Bioactive Aromatic Plant Extracts Modulate Metabolism and Inflammation in HeLa Cells
Source: Molecules. 2025 Nov 14;30(22):4401. doi: 10.3390/molecules30224401 (PMC12655313; doi:10.3390/molecules30224401)
Supplement: Supplementary file 1 [file molecules-30-04401-s001.zip › molecules-3961688-supplementary.docx]

Table S1 – Compounds identified by LC-MS for the ethanolic extracts of the different aromatic plants evaluated.

|  | **Compound name** | **Chemical formula** | **Mz** |
| --- | --- | --- | --- |
| ***Lemon verbena*** | | | |
| 1 | Acteoside | C_29_H_36_O_15_ | 623,196 |
| 2 | Eriodictyol | C_15_H_12_O_6_ | 287,056 |
| 3 | Apigenin | C_15_H_10_O_5_ | 269,046 |
| 4 | Luteolin | C_15_H_10_O_6_ | 285,04 |
| 5 | 2,4-dihydroxyheptadec-16-ynyl acetate | C_19_H_34_O_4_ | 325,238 |
| 6 | Cirsimaritin | C_17_H_14_O_6_ | 623,196 |
| 7 | Eupatilin | C_18_H_16_O_7_ | 343,08 |
| 8 | [(3S,4R,5S)-5-[(2S,3R,4S,5S,6R)-4,5-dihydroxy-6-(hydroxymethyl)-2-(4-hydroxyphenoxy)oxan-3-yl]oxy-3,4-dihydroxyoxolan-3-yl]methyl 4-hydroxybenzoate | C_18_H_16_O_7_ | 523,146 |
| 9 | (2Z)-4,6-dihydroxy-2-[(4-hydroxy-3,5-dimethoxyphenyl)methylidene]-1-benzofuran-3-one | C_17_H_14_O_7_ | 329,067 |
| 10 | Acacetin | C_16_H_12_O_5_ | 283,061 |
| 11 | Isoacteoside | C_29_H_36_O_15_ | 623,2 |
| 12 | Scutellarein-4 methy | C_16_H_12_O_6_ | 299,056 |
| 13 | 9-hydroxy-10,12-octadecadienoic aci | C_18_H_32_O_2_ | 295,228 |
| 14 | 7-Methoxyflavon | C_16_H_12_O_3_ | 267,066 |
| 15 | 7-hydroxy-2-(4-hydroxy-3,5-dimethoxyphenyl)-5-[(2S,3R,4S,5S,6R)-3,4,5-trihydroxy-6-(hydroxymethyl)oxan-2-yl]oxychromen-4-one | C_23_H_24_O_13_ | 491,12 |
| 16 | 6-Methoxyluteolin | C_16_H_12_O_7_ | 315,1 |
| 17 | (1S,4aS,7S,7aS)-7-hydroxy-7-methyl-1-[(2S,3R,4S,5S,6R)-3,4,5-trihydroxy-6-(hydroxymethyl)oxan-2-yl]oxy-4a,5,6,7a-tetrahydro-1H-cyclopenta[c]pyran-4-carboxylic acid | C_16_H_24_O_9_ | 375,127 |
| 18 | geniposidic acid | C_16_H_22_O_10_ | 373,114 |
| 19 | Naringenin | C_16_H_12_O_5_ | 271,1 |
| 20 | 3,3_,4_,7-TETRAHYDROXYFLAVONE | C_15_H_10_O_6_ | 301,071 |
| 21 | 5,7-dihydroxy-6-methoxy-2-[4-[(2S,3R,4S,5S,6R)-3,4,5-trihydroxy-6-(hydroxymethyl)oxan-2-yl]oxyphenyl]chromen-4-one | C_22_H_22_O_11_ | 461,109 |
| 22 | Isodeoxycholic acid | C_24_H_40_O_4_ | 783,577 |
| 23 | (2E)-N-[4-(acetylamino)phenyl]-3-(3,4-dimethoxyphenyl)prop-2-enamide | C_19_H_18_N_2_O_4_ | 385,141 |
| 24 | N-(2-methyl-3-oxo(2H,4H-benzo[3,4-e]1,4-oxazin-6-yl))(3,4,5-trimethoxyphenyl)c arboxamide | C_24_H_40_O_4_ | 342,239 |
| 25 | 4' 5 7-trihydroxy-3 6-dimethoxyflavone | C_17_H_14_O_7_ | 329,067 |
| 26 | Germanasim E | C_29_H_32_O_16_ | 475,123 |
| 27 | (2Z)-4,6-dihydroxy-2-[(4-hydroxy-3,5-dimethoxyphenyl)methylidene]-1-benzofuran-3-one | C_17_H_14_O_7_ | 329,067 |
| 28 | 3-methylquercetin | C_16_H_12_O_7_ | 315,051 |
| ***Thyme*** | | | |
| 1 | luteolin 4_-O-glucoside | C_21_H_20_O_11_ | 447,093 |
| 2 | Caffeic acid | C_9_H_8_O_4_ | 179,034 |
| 3 | Luteolin-7-O-glucoside | C_21_H_20_O_11_ | 447,093 |
| 4 | Luetolin | C_15_H_10_O_6_ | 285,04 |
| 5 | Dihydrokaempferol | C_15_H_12_O_6_ | 287,056 |
| 6 | Eriodictyol | C_15_H_12_O_6_ | 287,056 |
| 7 | rosmarinic acid | C_18_H_16_O_8_ | 575,1 |
| 8 | (+)-naringenin | C_15_H_12_O_5_ | 359,078 |
| 9 | cirsimaritin | C_17_H_14_O_6_ | 271,06 |
| 10 | Pindolol | C_14_H_20_N_2_O_2_ | 313,072 |
| 11 | Tricin | C_17_H_14_O_7_ | 265,146 |
| 12 | apigenin 6,8-digalactoside | C_27_H_30_O_15_ | 329,067 |
| 13 | (2Z)-4,6-dihydroxy-2-[(4-hydroxy-3,5-dimethoxyphenyl)methylidene]-1-benzofuran-3-one | C_17_H_14_O_7_ | 593,151 |
| 14 | Peonidin-3,5-O-di-beta-glucoside | C_28_H_33_O_16_^+^ | 329,067 |
| 15 | Eupalitin | C_17_H_14_O_7_ | 625,176 |
| 16 | 2,4-dihydroxyheptadec-16-ynyl acetate | C_19_H_34_O_9_ | 343,08 |
| 17 | skullcapflavone II | C_19_H_18_O_8_ | 325,238 |
| 18 | Diosmetin | C_16_H_12_O_6_ | 373,093 |
| 19 | Peonidin 3-O-glucoside | C_22_H_23_ClO_11_ | 463,101 |
| 20 | Isoquercetin | C_21_H_20_O_12_ | 463,089 |
| 21 | Jaceosidin | C_17_H_14_O_7_ | 327,218 |
| 22 | Taxifolin | C_15_H_12_O_7_ | 331,082 |
| 23 | (1r,3R,4s,5S)-4-{[(2E)-3-(3,4-Dihydroxyphenyl)-2-propenoyl]oxy}-1,3,5-trihydroxycyclohexanecarboxylic acid | C_16_H_18_O_9_ | 303,051 |
| 24 | Irigenin | C_18_H_16_O_8_ | 377,084 |
| 25 | 2,2,3,6-Tetramethoxychalcone | C_19_H_20_O_5_ | 329,143 |
| 26 | Quinic acid | C_7_H_12_O_6_ | 191,056 |
| 27 | (+)-7-epi-Syringaresinol 4'-glucoside | C_28_H_36_O_13_ | 579,209 |
| 28 | 5-methoxy-3-methyl-4-[(2S,3R,4S,5S,6R)-3,4,5-trihydroxy-6-[[(2R,3R,4S,5S,6R)-3,4,5-trihydroxy-6-(hydroxymethyl)oxan-2-yl]oxymethyl]oxan-2-yl]oxy-3H-benzo[f][2]benzofuran-1-one | C_26_H_32_O_14_ | 607,142 |
| ***Rosemary*** | | | |
| 1 | Luteolin-7-O-glucoside | C_21_H_20_O_11_ | 447,093 |
| 2 | luteolin 4_-O-glucoside | C_21_H_20_O_11_ | 447,093 |
| 3 | Apigenin-7-O-glucoside | C_21_H_20_O_10_ | 431,098 |
| 4 | Apigenin | C_15_H_10_O_5_ | 447,093 |
| 5 | Luetolin | C_15_H_10_O_6_ | 269,046 |
| 6 | Caffeic acid | C_9_H_8_O_4_ | 285,04 |
| 7 | Rosmarinic acid | C_18_H_16_O_8_ | 179,034 |
| 8 | Peonidin 3-O-glucoside | C_22_H_23_O_11_ | 359,077 |
| 9 | Hesperidin | C_28_H_34_O_15_ | 463,101 |
| 10 | cirsimaritin | C_17_H_14_O_6_ | 609,182 |
| 11 | Luteolin-7-glucuronide | C_21_H_18_O_12_ | 313,072 |
| 12 | Diosmetin-7-O-rutinoside | C_28_H_32_O_15_ | 607,167 |
| 13 | Tricin-5-glucoside | C_23_H_24_O_12_ | 491,12 |
| 14 | Furosemide | C_12_H_11_CIN_2_O_5_S | 329,001 |
| 15 | 3-phenyl-2-[(2S,3R,4S,5S,6R)-3,4,5-trihydroxy-6-[[(E)-3-(4-hydroxyphenyl)prop-2-enoyl]oxymethyl]oxan-2-yl]oxyprop-2-enoic acid | C_24_H_24_O_10_ | 471,13 |
| 16 | isorhamnetin-3-O-rutinoside | C_28_H_32_O_16_ | 623,162 |
| 17 | Scutellarein 4_-methyl ether | C_16_H_12_O_6_ | 299,056 |
| 18 | (2Z)-4,6-dihydroxy-2-[(4-hydroxy-3,5-dimethoxyphenyl)methylidene]-1-benzofuran-3-one | C_17_H_14_O_7_ | 329,067 |
| 19 | Quinic acid | C_7_H_12_O_6_ | 191,056 |
| 20 | 1-acetyl-3-{[2-(ethylpropyl)quinazolin-4-yl]amino}benzene | C_21_H_23_N_3_O_6_ | 332,177 |
| 21 | 5,7-dihydroxy-2-(4-hydroxyphenyl)-3,6-dimethoxy-4H-chromen-4-one | C_17_H_14_O_7_ | 329,066 |
| 22 | 7-hydroxy-2-(4-hydroxy-3,5-dimethoxyphenyl)-5-[(2S,3R,4S,5S,6R)-3,4,5-trihydroxy-6-(hydroxymethyl)oxan-2-yl]oxychromen-4-one | C_23_H_24_O_12_ | 491,12 |
| 23 | Acacetin | C_16_H_12_O_5_ | 283,061 |
| 24 | Peonidin-3-O-beta-galactoside | C_22_H_22_O_11_ | 461,109 |
| 25 | (1R,2R,4aS,6aS,6bR,10S,12aR,14bS)-1,8,10-trihydroxy-1,2,6a,6b,9,9,12a-heptamethyl-2,3,4,5,6,6a,7,8,8a,10,11,12,13,14b-tetradecahydropicene-4a-carboxylic acid | C_30_H_48_O_5_ | 487,343 |
| 26 | Kaempferol-7-O-neohesperidoside | C_27_H_30_O_15_ | 593,151 |
| 27 | Diosmetin | C_16_H_12_O_6_ | 299,056 |
| 28 | 9-methoxy-7-[4-[(2S,3R,4S,5S,6R)-3,4,5-trihydroxy-6-(hydroxymethyl)oxan-2-yl]oxyphenyl]-[1,3]dioxolo[4,5-g]chromen-8-one | C_23_H_22_O_11_ | 475,123 |
| 29 | Luteolin-7,3_-di-O-glucoside | C_27_H_30_O_16_ | 609,14 |
| 30 | Biochanin-7-O-glucoside | C_22_H_22_O_10_ | 447,129 |
| 31 | Viscidulin III | C_17_H_14_O_8_ | 345,062 |
| 32 | 6-Methoxyluteolin | C_16_H_12_O_7_ | 315,1 |
| 33 | beta-D-Glucopyranoside, 2-hydroxy-4-(2-propen-1-yl)phenyl 6-O-beta-D-glucopyranosyl- | C_21_H_30_O_12_ | 519,172 |
| 34 | (+)-syringaresinol beta-D-glucoside | C_28_H_36_O_13_ | 579,209 |
| 35 | 9-methoxy-7-[4-[3,4,5-trihydroxy-6-[[3,4,5-trihydroxy-6-(hydroxymethyl)oxan-2-yl]oxymethyl]oxan-2-yl]oxyphenyl]-[1,3]dioxolo[4,5-g]chromen-8-one | C_29_H_32_O_16_ | 637,176 |
| 36 | Iridin | C_24_H_26_O_13_ | 523,145 |
| 37 | [7-(3,4-dihydroxyphenyl)-1-(4-hydroxyphenyl)heptan-3-yl] acetate | C_21_H_26_O_5_ | 357,171 |
| 38 | Eriodictyol | C_15_H_12_O_6_ | 285,039 |
| 39 | syringaresinol | C_22_H_26_O_8_ | 417,155 |
| 40 | Isorhamnetin | C_16_H_12_O_7_ | 317 |
| 41 | Sayaendoside | C_19_H_28_O_10_ | 415,161 |
| 42 | (2R,3S,4S,5R,6R)-5-[(2S,3R,4R)-3,4-dihydroxy-4-(hydroxymethyl)oxolan-2-yl]oxy-2-(hydroxymethyl)-6-(2-phenylethoxy)oxane-3,4-diol | C_19_H_28_O_10_ | 415,161 |
| 43 | 6-hydroxy-3-[3-hydroxy-4-[3,4,5-trihydroxy-6-[[3,4,5-trihydroxy-6-(hydroxymethyl)oxan-2-yl]oxymethyl]oxan-2-yl]oxyphenyl]-5,7-dimethoxychromen-4-one | C_29_H_34_O_17_ | 653,172 |
| 44 | Paederoside | C_18_H_22_O_11_S | 445,081 |
| ***Costemary*** | | | |
| 1 | Apigenin | C_15_H_10_O_5_ | 269,046 |
| 2 | Caffeic acid | C_9_H_8_O_4_ | 179,034 |
| 3 | 2-(3,4-dihydroxyphenyl)-5-hydroxy-7-[(2S,3R,4S,5S,6R)-3,4,5-trihydroxy-6-[[(2R,3R,4R,5R,6S)-3,4,5-trihydroxy-6-methyloxan-2-yl]oxymethyl]oxan-2-yl]oxychromen-4-one | C_27_H_30_O_15_ | 593,151 |
| 4 | Luteolin-7-O-glucoside | C_21_H_20_O_11_ | 447,093 |
| 5 | Rosmarinic acid | C_18_H_16_O_8_ | 719,2 |
| 6 | Neochlorogenic Acid | C_16_H_18_O_9_ | 353,088 |
| 7 | Rhoifolin | C_27_H_30_O_14_ | 577,152 |
| 8 | Eriodictyol-7-O-rutinoside | C_27_H_32_O_15_ | 595,167 |
| 9 | Luteolin | C_15_H_10_O_6_ | 285,04 |
| 10 | (1r,3R,4s,5S)-4-{[(2E)-3-(3,4-Dihydroxyphenyl)-2-propenoyl]oxy}-1,3,5-trihydroxycyclohexanecarboxylic acid | C_16_H_18_O_9_ | 353,088 |
| 11 | Eriodictyol | C_15_H_12_O_6_ | 287,056 |
| 12 | Luteolin 7-glucuronide | C_21_H_18_O_12_ | 461,088 |
| 13 | Cosmosiine | C_21_H_20_O_10_ | 431,098 |
| 14 | Calceolarioside A | C_23_H_26_O_11_ | 477,14 |
| 15 | Isoquercetin | C_21_H_20_O_12_ | 464,38 |
| 16 | Hesperetin | C_16_H_14_O_6_ | 301,072 |
| 17 | Kaempferol-3-O-glucuronoside | C_21_H_18_O_12_ | 461,073 |
| 18 | rac Hesperidine | C_28_H_34_O_15_ | 609,182 |
| 19 | Eupatilin | C_18_H_16_O_7_ | 343,08 |
| 20 | Skullcapflavone II | C_19_H_18_O_8_ | 373,093 |
| 21 | Tiliroside | C_30_H_26_O_13_ | 1187,27 |
| 22 | (2Z)-4,6-dihydroxy-2-[(4-hydroxy-3,5-dimethoxyphenyl)methylidene]-1-benzofuran-3-one | C_17_H_14_O_7_ | 329,067 |
| 23 | (2R)-2-[(E)-3-[3-[(1R)-1-carboxy-2-(3,4-dihydroxyphenyl)ethoxy]carbonyl-2-(3,4-dihydroxyphenyl)-7-hydroxy-2,3-dihydro-1-benzofuran-4-yl]prop-2-enoyl]oxy-3-(3,4-dihydroxyphenyl)propanoic acid | C_36_H_30_O_16_ | 717,146 |
| 24 | 1-(3,4-dihydroxyphenyl)-6,7-dihydroxy-1,2-dihydronaphthalene-2,3-dicarboxylic acid | C_18_H_14_O_8_ | 357,062 |
| 25 | Irigenin | C_18_H_16_O_8_ | 359,077 |
| 26 | Diosmin | C_28_H_32_O_15_ | 607,167 |
| 27 | Okanin-4’-O-glucoside | C_21_H_22_O_11_ | 449,109 |
| 28 | DOCOSANOL | C_22_H_46_O | 325,348 |
| 29 | Lithospermic acid | C_27_H_22_O_12_ | 537,104 |
| 30 | Loganic acid | C_16_H_24_O_10_ | 375 |
| 31 | Kaempferol-7-O-neohesperidoside | C_27_H_30_O_15_ | 595,1 |
| 32 | Luteolin-7,3_-di-O-glucoside | C_27_H_30_O_16_ | 609,14 |
| 33 | Vanillic acid + O-sulfonateHex | C_14_H_18_O_12_S | 409,044 |
| 34 | 1-O-[(2alpha,3beta,5xi,6beta,9xi,18xi)-2,3,6,23-Tetrahydroxy-28-oxoolean-12-en-28-yl]-beta-D-glucopyranose | C_36_H_58_O_11_ | 711,396 |
| 35 | Pagoside | C_24_H_28_O_11_ | 491,156 |
| 36 | Eriodictyol-7-O-glucoside | C_21_H_22_O_11_ | 449,109 |
| 37 | 01!N-[2-(4-(4-Chlorophenyl)piperazin-1-yl)ethyl]-3-methoxybenzamide | C_20_H_24_O_2_ | 374,166 |
| 38 | Betanine | C_24_H_26_N_2_O_13_ | 551,158 |
